# Morphological changes in plasma-exposed poultry red mites (*Dermanyssus gallinae*) using high-resolution video camera and optical coherence tomography (OCT)

**DOI:** 10.1007/s10493-024-00934-3

**Published:** 2024-06-27

**Authors:** Vanessa Rüster, Henrik Werner, Georg Avramidis, Stephan Wieneke, Christina Strube, Christian Schnabel, Thomas Bartels

**Affiliations:** 1 Institute for Parasitology, Centre for Infection Medicine, University of Veterinary Medicine Hannover, Hannover, Germany; 2 Institute of Animal Welfare and Animal Husbandry, Friedrich-Loeffler-Institut, Celle, Germany; 3 Faculty of Engineering and Health, University of Applied Sciences and Arts, Göttingen, Germany; 4 Departement of Anesthesiology and Intensive Care Medicine, Clinical Sensoring and Monitoring, Faculty of Medicine, Technische Universität Dresden, Dresden, Germany

**Keywords:** Plasma-based pest management, Acaricidal effects, Mode of action, Alternative acaricides, Optical coherence tomography

## Abstract

*Dermanyssus gallinae*, the poultry red mite (PRM), is a hematophagous temporary ectoparasite that causes serious economic losses and animal health impairment on laying hen farms worldwide. Control is limited by the parasite’s hidden lifestyle, restrictions on the use of chemical acaricides and the development of resistance against certain drug classes. As a result, research was conducted to explore alternative control methods. In recent years, atmospheric pressure plasma has been increasingly reported as an alternative to chemical acaricides for pest control. This physical method has also shown promising against PRM under laboratory conditions. However, the detailed mechanisms of action have not yet been elucidated. In the present study, the effects of cold atmospheric pressure plasma on PRM were investigated using digital videography and optical coherence tomography (OCT), an imaging technique that visualizes the topography of surfaces and internal structures. Digital videography showed that a redistribution of the contents of the intestinal tract and excretory organs (Malpighian tubules) occurred immediately after plasma exposure. The body fluids reached the distal leg segments of PRM and parts of the haemocoel showed whiter and denser clumps, indicating a coagulation of the haemocoel components. OCT showed a loss of the boundaries of the hollow organs in transverse and sagittal sectional images as well as in the three-dimensional image reconstruction. In addition, a dorso-ventral shrinkage of the idiosoma was observed in plasma-exposed mites, which had shrunk to 44.0% of its original height six minutes after plasma exposure.

## Introduction

The poultry red mite (PRM) *Dermanyssus gallinae* is a hematophagous temporary ectoparasite affecting birds of various species. Particularly it is prevalent in laying hen husbandry (Flochlay et al. 2017; Nunn et al. 2020). An infestation not only significantly reduces the health and welfare of host animals and personnel, but also causes immense economic damage. Environmental chemical acaricides and acaricidal drugs are mainly used to control PRM infestations (Sommer 2011; Decru et al. 2020). However, some of them are considered carcinogenic, contain neurotoxic substances and have effects on mental health (Ansari et al. 2014; Soulié et al. 2021). Additionally, the use of acaricides is restricted by regulatory requirements and stricter legislation (Decru et al. 2020). Furthermore, the use of chemical acaricides has been increasingly restricted in recent decades due to the increasing resistance of PRM. Resistance to products containing organophosphates, carbamates and pyrethroids has been reported (Liebisch and Liebisch 2003; Marangi et al. 2009; Abbas et al. 2014). Therefore, physical and biological control methods have gained importance (Lima-Barbero et al. 2020).

In recent years, intensive research has been conducted on the development of effective and sustainable methods of controlling PRM infestations. Laboratory experiments and field studies have investigated the use of temperature, showing the effectiveness of heating the hen house to over 45 °C or using hot water high-pressure cleaners (Decru et al. 2020). Lighting programmes influence the behavior of PRM and can reduce the population under laboratory conditions (Zoons 2004; Stafford et al. 2006). However, changes in light regimes may affect animal welfare, and national guidelines for livestock must be observed (Mul and Koenraadt 2009). Diatomaceous earth or synthetic amorphous silica are common biophysical measures against PRM (Maurer and Perler 2006; Kilpinen and Steenberg 2009; Schulz et al. 2014). Ulrichs et al. (2020) demonstrated an efficient control of PRM in a hen house for 46 weeks using silica-based products. In addition, repellent essential oils and plant-based preparations showed promising efficacy in vitro, but had limited success in the field (Kim et al. 2004; Maurer et al. 2009; Locher 2010; Faghihzadeh et al. 2014). Natural enemies such as predatory mites, bacteria and fungi can also be used against the PRM (Nordenfors and Höglund 2000; Roy et al. 2017; Alves et al. 2023), but such biological methods are rather supportive measures as they are significantly influenced by factors like the temperature (Ali et al. 2012; Lesna et al. 2012; Hwang 2023). Novel physical control methods such as high-voltage pulses (Ueno et al. 2023) have shown high efficacy against PRM in experimental studies. Obviously, there is still a need to explore alternative, sustainable and effective control strategies for the field (Hwang 2023).

A possible solution with great potential for effective and sustainable control could also be integrated pest management (IPM) as reported recently (Rüster et al. 2023). IPM involves the combination of preventive measures, monitoring techniques and effective control methods of varying efficacy (Sparagano et al. 2014). The main aim of this approach is to reduce the use of environmental chemical acaricides and acaricidal drugs, which should only be used when alternative methods have not been sufficiently effective (Mul 2017). Therefore, IPM could limit the use of pesticides and reduce or minimize the side effects and risks to human health and the environment (FAO 2020; Hwang 2023).

In recent years, promising studies have been reported on the application of cold atmospheric pressure plasma (CAPP) to several arthropod pests. Physical plasma is already being used in agriculture for seed treatment, crop decontamination, soil remediation and control of various insect pests (Zhang et al. 2014; Ohta 2016; Brandenburg et al. 2019; Carpen et al. 2019; ten Bosch 2022). The red flour beetle (*Tribolium castaneum*), the Indian meal moth (*Plodia interpunctella*) and the head lice (*Pediculus humanus capitis*) can be effectively killed with non-thermal plasma (ten Bosch et al. 2019; Nasr et al. 2020; Sayed et al. 2021). Short-term exposure to CAPP also resulted in effective control of all developmental stages of PRM, including mite eggs (Rüster et al. 2022). Irrespective of the setting parameters (electrical power and exposure time) used, plasma-exposed eggs were unable to develop, i.e. no larval hatch was observed in any of the eggs. In the unexposed control group, normally developed larvae hatched from all mite eggs. After treatment with 10 W power and an exposure time of 1.0 s, high mortality was observed immediately after plasma exposure in larvae (96.6%), protonymphs (92.2%), deutonymphs (85.5%) and adult males (93.3%), while mortality in adult females (42.2%) was initially much lower. However, almost all initially surviving mites died within 60 min of plasma exposure, only a few protonymphs died up to 120 min after plasma exposure (Rüster et al. 2022). However, the mode of action of plasma on mite species is still unknown. To determine the lethal effects of plasma, exposed PRM were examined using optical coherence tomography and a high-resolution video camera. This allows repeated non-contact imaging of the treated PRM to visualize the short-term changes in morphological structures as a result of plasma treatment.

## Materials and methods

### Poultry red mites

Mites originated from the acaricide-susceptible *D. gallinae* F0 isolate maintained at the Institute for Parasitology, University of Veterinary Medicine Hannover. This isolate was kept in breeding boxes at room temperature. For feeding, a hen was placed in the box at weekly or biweekly intervals and the mites were allowed to take a blood meal. For the present study, non-engorged (i.e., grey), alive adult females were collected and subjected to examinations within 2 days of collection.

### Plasma setup and treatment of PRM

Exposure to cold atmospheric pressure plasma (CAPP) was performed using a dielectric barrier discharge (DBD) system using the Tantec generator V-X20 (Tantec, Lunderskov, Denmark) as described by Rüster et al. (2022). CAPP was produced at room temperature and under atmospheric pressure in ambient air (composition: nitrogen 78.08%, oxygen 20.95%, argon 0.93%, carbon dioxide < 0.04%, small and variable amounts of trace gases, e.g. methane, ozone, various noble gases; Schlesinger and Bernhardt 2020), which was generated between two parallel electrodes (discharge distance = 1 mm) in a capacitor arrangement. In preliminary tests, a power of 30 W and an exposition time of 3.0 s proved to be suitable for the intended imaging. The electrode discharge area was 22 cm^2^, resulting in power density of 1.36 W/cm^2^. For the imaging studies, ten alive adult female mites were individually attached on the ground electrode of the DBD with double-sided adhesive tape using a fine brush to restrict mite movement.

### Digital videography and optical coherence tomography (OCT)

**Fig. 1:**
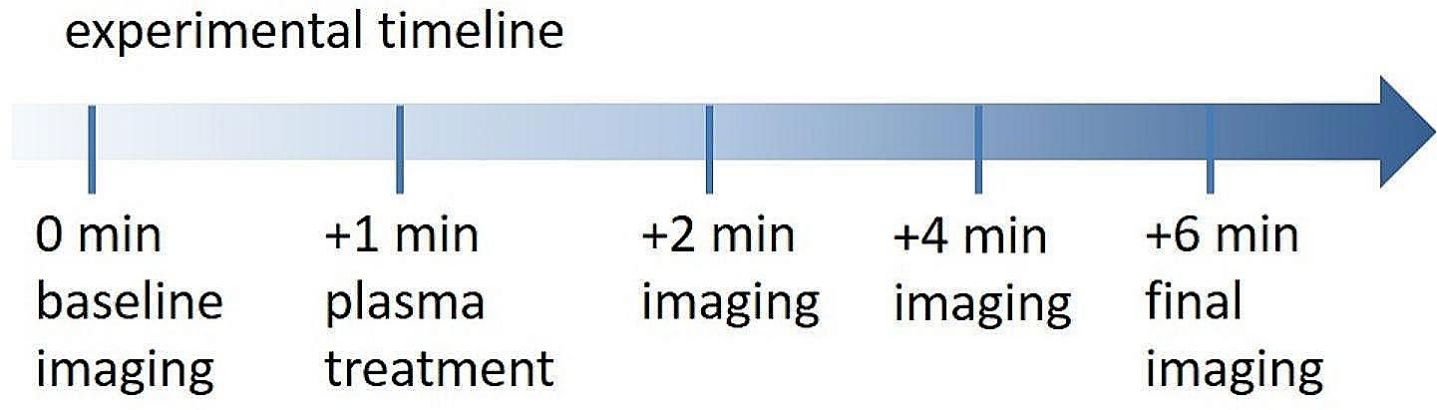
Timeline of the experiment: Starting with a baseline scan, plasma treatment after one minute, followed by imaging every two minutes until the final imaging after six minutes

Ten adult female PRM were initially examined by OCT and videography at the observation time (t_obs_) of 1 min before CAPP exposure (t_obs_ = 0:00 min). Thus, each mite served as its own untreated control. After exposure to CAPP at t_obs_ = 2:00 min, t_obs_ = 4:00 min and t_obs_ = 6:00 min, the mites were re-examined to observe morphological changes and thus possible effects of the plasma treatment. The timeline of the experiment is shown in Fig. 1.

Live imaging of plasma treated mites with OCT and videography were performed at the department of Clinical Sensoring and Monitoring at the Medical Faculty of the Technische Universität Dresden, Germany. Optical coherence tomography (OCT) is an interferometric imaging technique based on near-infrared light that allows contactless and marker-free visualization of the inner structure of scattering materials like biological tissue with a spatial resolution in the micrometer range (Schnabel et al. 2013, 2014; Jannasch et al. 2021).

**Fig. 2:**
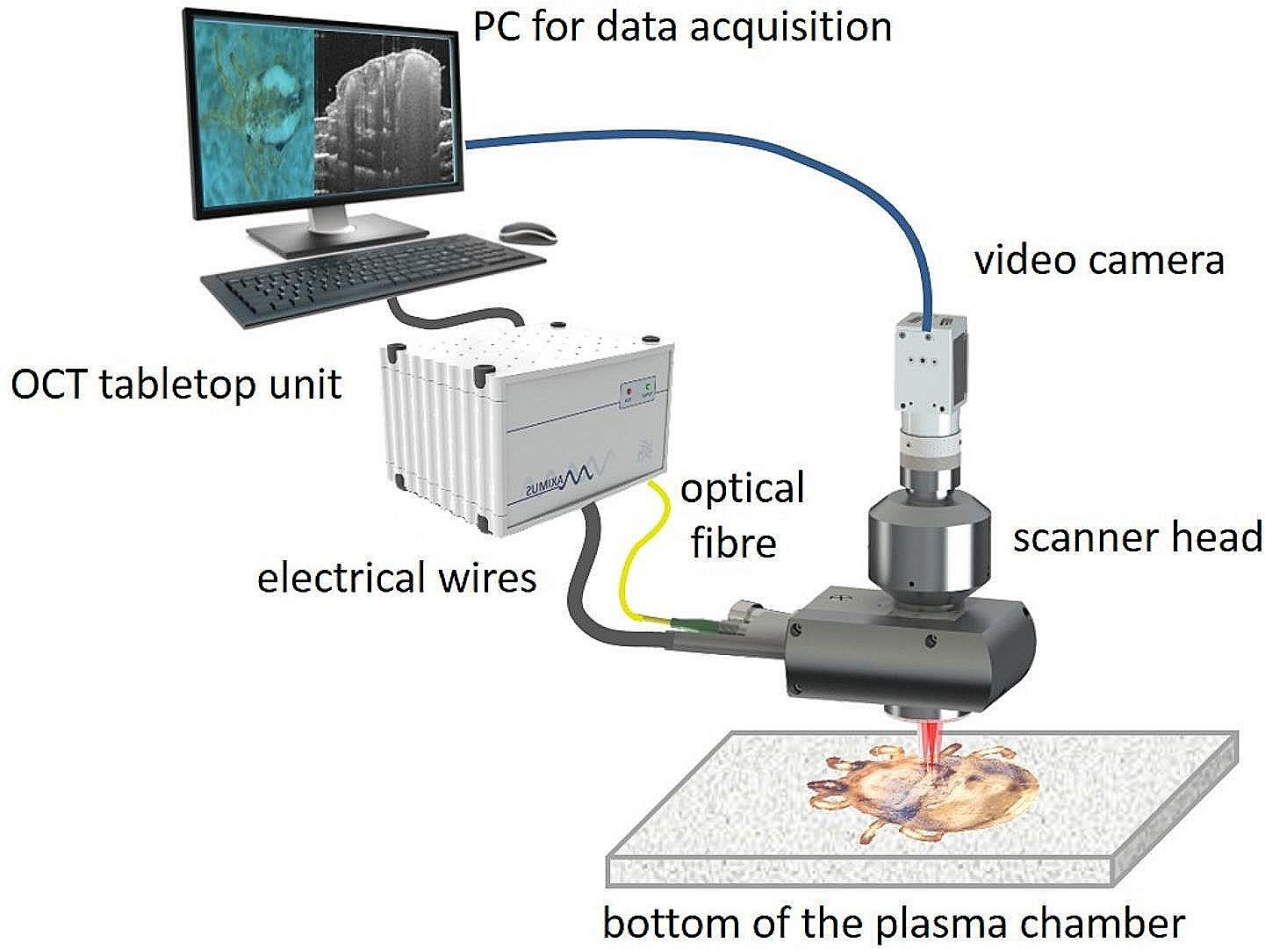
Imaging setup of the optical coherence tomography (OCT) system and the high-resolution video camera

The OCT system used in this study consists of a custom build spectrometer and scanner head containing a Michelson interferometer as described in Schnabel et al. 2014 (Fig. 2).

OCT is used to obtain a large number of sectional images that can then be analyzed using the Fiji program, an open source image processing package based on ImageJ2 (Schindelin et al. 2012). First, the OCT data stack is exported as an uncompressed.avi file. Care must be taken to ensure correct scaling so that the distance between voxels is uniform in all three dimensions matching the real morphologic scale (voxel distance ≙ 2.33 μm). The number of sectional images varied depending on the size of the mite. To measure the idiosoma of the mite, it was displayed in orthogonal projection and the center of the mite was determined. The sagittal, transverse and horizontal views were saved as separate files. To measure the length, a line was drawn from the cranial to the caudal end of the mite in the sagittal view. In the center of the length, the height of the mite was determined by a vertical line. Similar procedure was used to measure the width. The center of the mite was measured in cross-section from left to right.

### Statistical analysis

The idiosoma dimension data were analyzed using Dunnett’s multiple comparisons test. A *p*-value ≤ 0.05 was considered statistically significant. All analyses were performed using GraphPad Prism (version 6, GraphPad Software, La Jolla, CA, USA).

## Results

### Video analysis

**Fig. 3:**
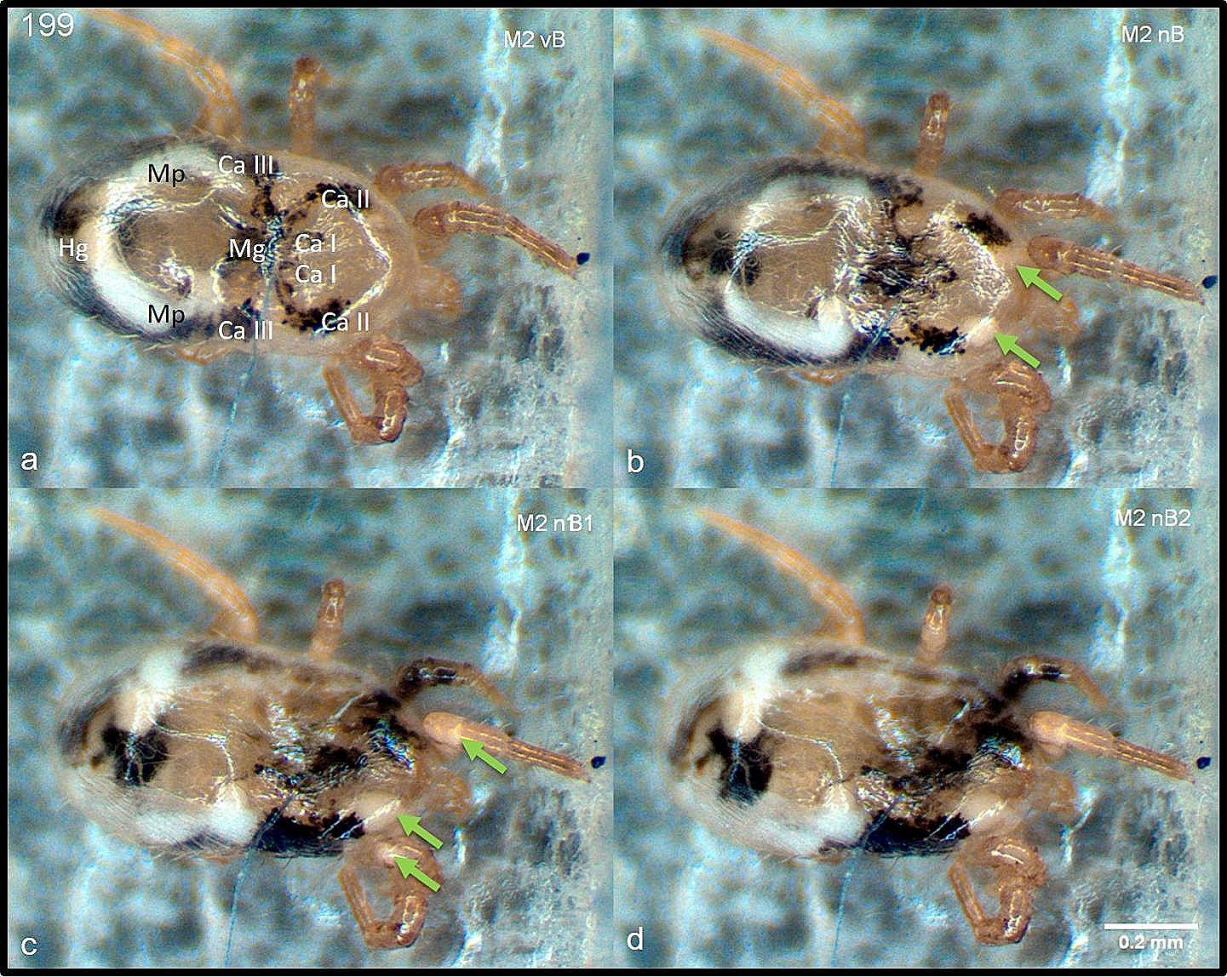
Comparison of video sequence images of a female *D. gallinae* individual. (**a**) Untreated living mite before CAPP exposure (t_obs_ = 0:00 min), (**b**) living mite after plasma exposure (t_obs_ = 2:00 min), (**c**) inanimate mite after plasma exposure (t_obs_ = 4:00 min) and (**d**) inanimate mite min after exposure (t_obs_ = 6:00) to CAPP at 30 W for 3.0 s. Arrows depict the white, denser haemocoel. Ca I-III– caeca IIII, Mg– midgut, Mp– Malpighian tubules, Hg– hindgut. Bar = 0.2 mm

Video recordings of each mite were taken one minute before plasma exposure (t_obs_ = 0:00 min) until five minutes after plasma exposure (t_obs_ = 6:00 min). Image sections of these video sequences before plasma exposure at t_obs_ = 0:00 min and after plasma exposure at t_obs_ = 2:00 min, t_obs_ = 4:00 min and t_obs_ = 6:00 min are shown in Fig. 3. The video recordings showed a redistribution of the white milky contents of the Malpighian tubules into the anterior part of the mite body starting immediately after plasma exposure. In addition, a movement in the intestinal tubules was observed immediately after plasma exposure, showing a flow of dark red blood residues from the hindgut towards the midgut up to the anterior part of the mite (Fig. 3c, d). In addition, blood residues and contents of the Malpighian tubules were found in the anterior segments of the legs after plasma exposure (Fig. 3c, d). In addition, parts of the hemolymph clumped, appearing whiter and denser after plasma treatment (Fig. 3a, b; light green arrows).

### OCT analysis

**Fig. 4:**
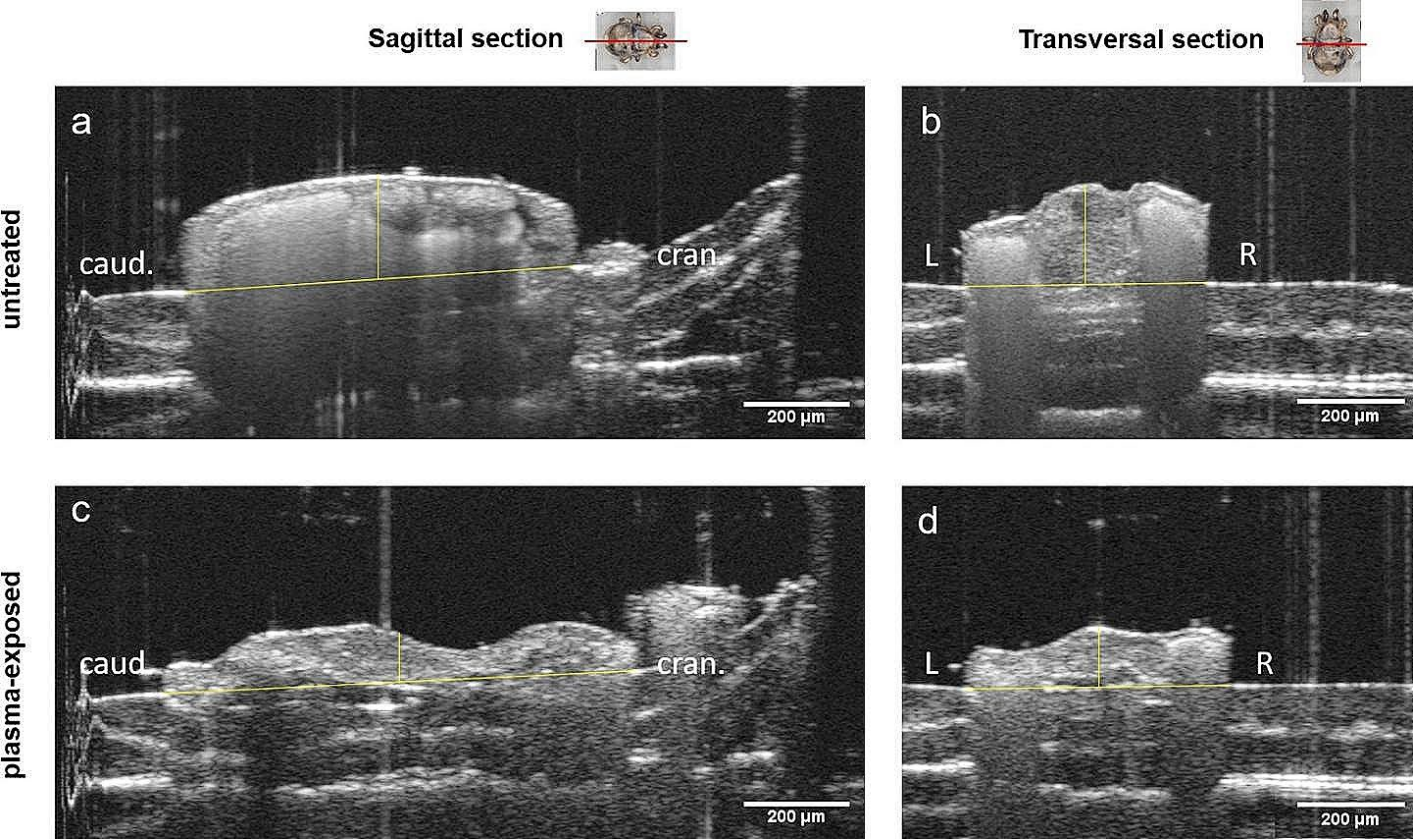
Sagittal and transverse sections of a female *D. gallinae* individual by OCT. (**a, b**): Untreated mite (t_obs_ = 2:00 min) and (**c, d**): mite after plasma exposure at 30 W and 3.0 s (t_obs_ = 6:00 min) showing loss of the organ-enclosing structures and flattened height of the idiosoma. Bar = 200 μm

**Fig. 5:**
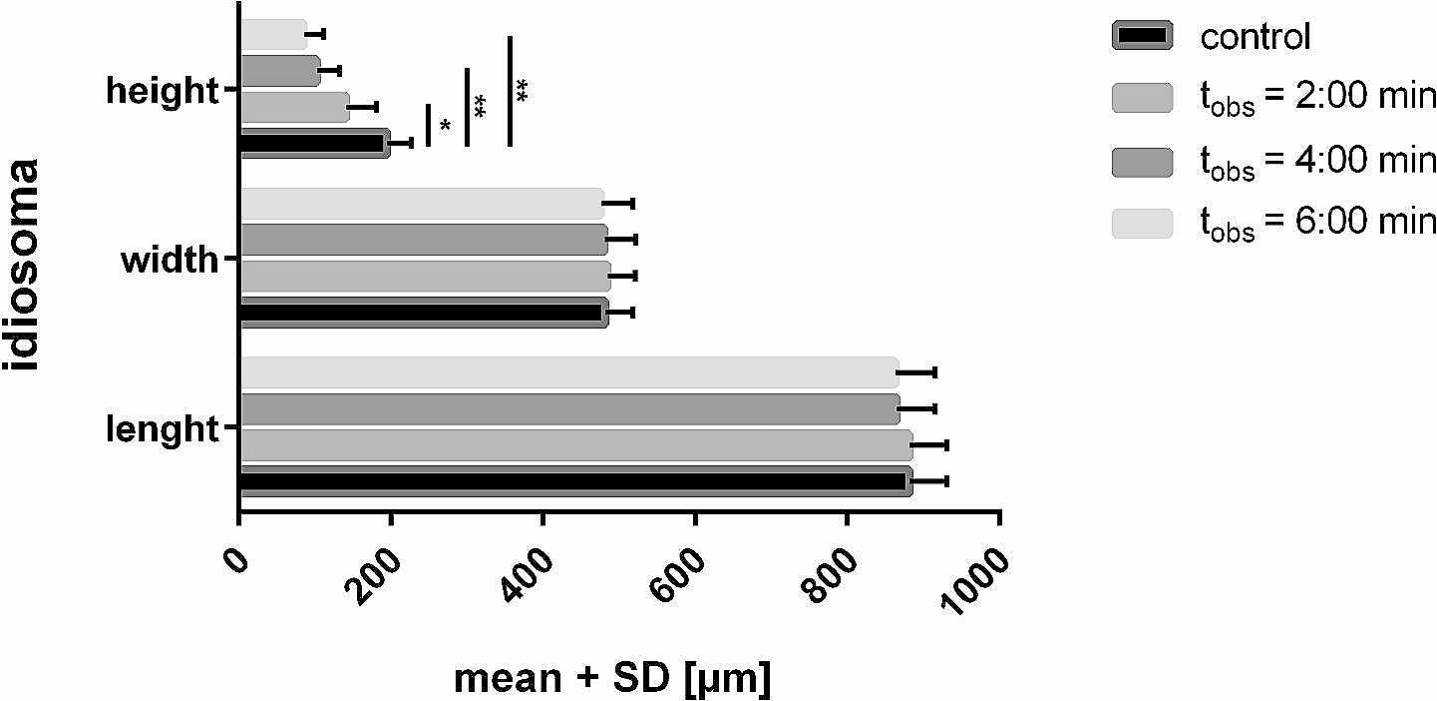
Comparison of the mean idiosoma dimensions of ten female *D. gallinae* individuals before (control, t_obs_ = 0:00 min) and after plasma exposure at 30 W for 3.0 s. Bars are presented as mean + standard deviation [µm]. Plasma-exposure has significantly flattened the mites (Dunnett’s multiple comparisons test; * *p* < 0.005; ** *p* < 0.0001)

**Fig. 6:**
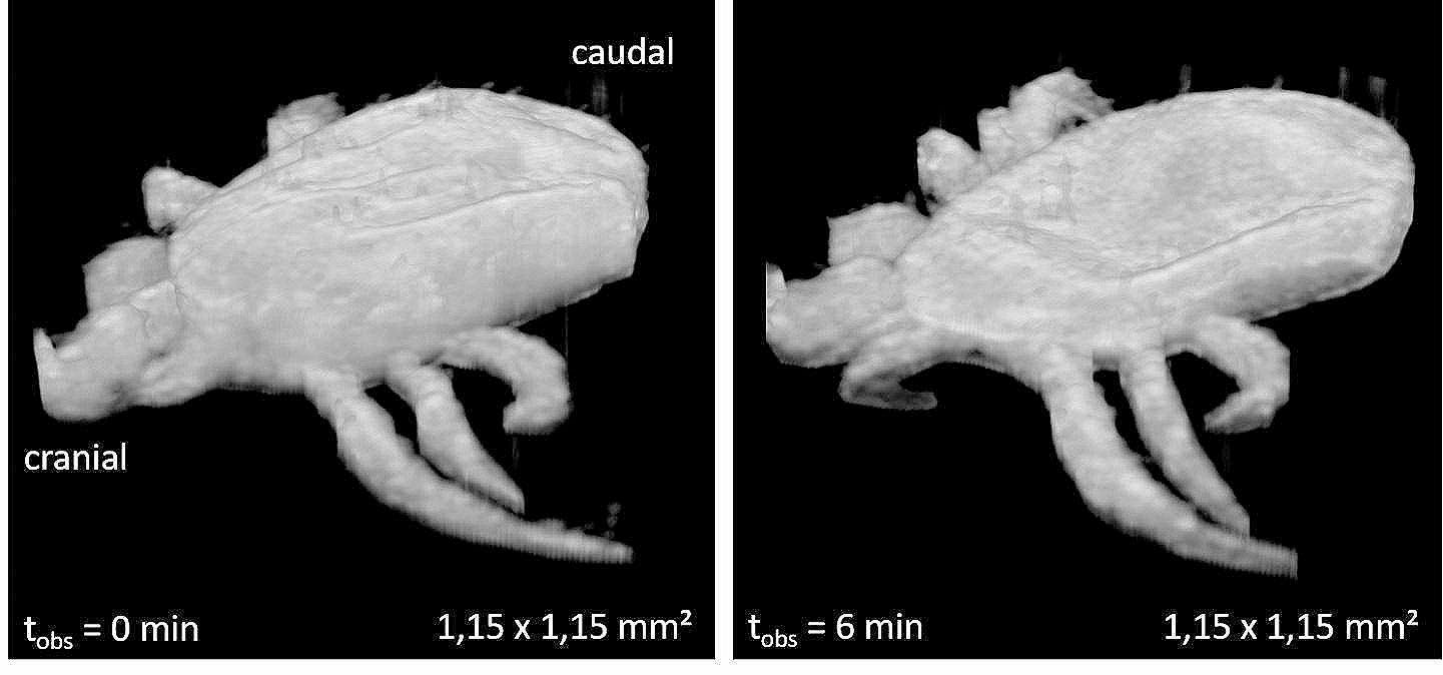
3D reconstruction of a female *D. gallinae* mite before (t_obs_ = 0:00 min) and after (t_obs_ = 6:00 min) plasma exposure at a power of 30 W for 3.0 s (mite covered with a fine brush hair)

After exposure to CAPP, real-time OCT imaging showed reduced contrast of the internal organs. The clear borders of the Malpighian tubules, which were previously visible, disappeared after exposure to CAPP (Fig. 4). A subsidence of the idiosoma was observed immediately after CAPP irradiation. This effect became more pronounced with time (Fig. 5). A significant reduction in idiosoma height of the plasma-exposed mites to 72.4 ± 19.2%, 52.7 ± 13.5% and 44.0 ± 12.7% compared to the pre-treatment control (t_obs_ = 0:00 min) was recorded for t_obs_ = 2:00 min, t_obs_ = 4:00 min and t_obs_ = 6:00 min, respectively (Fig. 5). In contrast, the measurements of length and width showed only minimal changes (Fig. 5). At the time t_obs_ = 2:00 min and t_obs_ = 4:00 min, no changes in width could be detected, while mites were reduced in width by 1.1% at t_obs_ = 6:00 min. The length of the mites was reduced by 1.9% at t_obs_ = 4:00 min and by 2.1% at t_obs_ = 6:00 min. The shrinkage effect from t_obs_ = 0:00 min to t_obs_ = 6:00 is clearly visible in the 3D OCT reconstruction (Fig. 6).

## Discussion

The development of resistance to chemical acaricides is a serious problem in the management of PRM infestations (Lima-Barbero et al. 2020; Decru et al. 2020). Nocturnal activity, hiding in places that are difficult to access, and survival of long periods of starvation of up to 34 weeks make PRM difficult to control effectively and sustainably (Nordenfors and Höglund 2000; Kirkwood 1963). Mite control is not only limited by the hidden lifestyle of PRM, but effective control is also limited by the risk of acaricide residues in eggs and meat. As a result, the use of environmental chemical acaricides and acaricidal drugs is severely restricted by strict legislation (Decru et al. 2020). Moreover, consumer demand for pesticide-free products is increasing (Mul 2013), so there is a great need to develop effective and sustainable control methods.

Different studies indicate that CAPP has a killing effect on a wide range of microorganisms and arthropods (Donohue et al. 2006; ten Bosch et al. 2019; Sayed et al. 2021; Rüster et al. 2022; Anuntagool et al. 2023). The potentially inactivating components formed in the plasma can include charged particles, UV radiation, ozone and reactive species (Matthes et al. 2013). However, the exact mode of action of CAPP on microorganisms and arthropods is not well understood. In the present study, high resolution video sequence analysis and OCT showed that morphological changes occurred inside the examined female *D. gallinae* mites after exposure to CAPP. Like light microscopy, OCT has a resolution in the micrometer range. However, the advantage of OCT is that it can visualize the internal structures of a sample like ultrasonic imaging (Nawaz et al. 2023). In addition, no sample preparation is required, which means that OCT can be used repeatedly on living biological material (Berry 2022). This fast and non-contact method is widely used in medicine to visualize structures of the eye (Berry 2022; Lu et al. 2023). In recent years, it has also proved useful in entomology. OCT has been used to visualize the brain of honey bees (*Apis mellifera*), the heart of the fruit fly *Drosophila melanogaster* and the internal structures of the Indian meal moth (*Plodia interpunctella*) during its developmental cycle (Bizheva et al. 2004; Choma et al. 2006; Wolf et al. 2006). OCT has also been shown to be a suitable tool for phenotypic analysis in embryonic development of insects (Su et al. 2020). Similarly, the process of light adaptation in moths has been studied by visualizing the anatomical structures of the compound eye (Berry 2022). Our investigations showed a redistribution of the gut content up to the pretarsal segments of the limbs. Similarly, a displacement of the contents of the Malpighian tubules into the surrounding haemocoel was observed, suggesting a rupture of organ-confining structures. Similar findings were observed in plasma-exposed head lice (*Pediculus humanus capitis*) in OCT, showing a rupture between the midgut and the head, resulting in internal damage to the intestine. The lice showed leakage of digested blood from the ventricle into the surrounding hemolymph, which spread to the legs (ten Bosch et al. 2019; Bosch et al. 2022). In German cockroaches (*Blattella germanica*), exposure to high doses of plasma resulted in abdominal ruptures (Donohue et al. 2008). The cracks could be caused by the plasma-damaged walls of the organs in combination with the plasma- and/or current-induced heating or heating due to dielectric losses and the resulting increase in pressure inside the mite. The pressure destroys the walls of the internal hollow organs, allowing their contents to spill out. In PRM, the structure-limiting membranes are no longer separable after plasma treatment, which may also be caused by a change in the permeability of the peritrophic membrane. Plasma exposure leads to the destruction of internal organs, resulting in the distribution of intestinal contents and contents of the Malpighian tubules in the haemocoel. The PRMs died within the observation period. It is likely that digestive enzymes are released, leading to autolysis of the mite.

The whitish altered and denser hemolymph of the mites exposed to plasma in our study suggests that parts of the hemolymph were coagulated. The changes in the haemocoel could be due to the coagulation of proteins. These findings are similar to the coagulation of the hemolymph due to oxidative stress in cold plasma-treated red flour beetles (*Tribolium castaneum*) (Ramanan et al. 2018), but local thermal effects might also play a role. Free radicals produced by reactive oxygen species also lead to indirect effects on the DNA, immune system and circulation of insects (Sayed et al. 2021; Ji et al. 2019). In addition, CAPP affected the neuromuscular system of insects through surface electrostatic effects, causing ataxia, loss of vibrotaxis, phototaxis and thigmotaxis (Donohue et al. 2006).

In vivo imaging by OCT allowed real-time detection of the mite body shrinkage immediately after plasma exposure. The plasma-exposed mites appeared desiccated. Dehydration after plasma exposure has previously been observed in German cockroaches (Donohue et al. 2008) and head lice (ten Bosch et al. 2019). In addition to the direct and indirect thermal effects of plasma described above, Donohue et al. (2008) proposed that dehydration can be caused by the breakdown of C-H bonds in the lipid layer of the insect cuticle, which ultimately leads to death. Similarly, exposure to cold plasma resulted in body malformation in red flour beetles (Ramanan et al. 2018) and head lice (ten Bosch et al. 2019). The shrinkage could be due to water loss as a result of increased respiratory or metabolic rates, probably due to the effects on the nervous and neuromuscular systems (Donohue et al. 2006). These desiccation effects can also be observed after the use of inert dusts due to the destruction of the protective wax layer of the mite cuticle, resulting in increased cuticle permeability and fluid loss (Ebeling 1971). Dorsally and ventrally, the idiosoma is stabilised by sclerotized shields (dorsal: dorsal shield; ventral: sternal shield, genitoventral shield, anal shield), presumably making it more resistant to changes in length and width than to changes in height.

PRM is usually controlled with environmental chemical acaricides or acaricidal drugs (Sommer 2011; Decru et al. 2020). The use of pesticides should be conscientious and minimized to reduce the impact on animals, humans and the environment (Decru et al. 2020). In addition, this could also help to prevent the development of resistance and prolong the availability of effective acaricides. Effective long-term methods against PRM are needed to minimize high control costs, animal losses and production losses. There is an urgent need for alternative control methods due to the development of resistance to chemical acaricides and increasing consumer demand for chemical-free food (Soulié et al. 2021). CAPP has shown promising in vitro acaricidal activity (Rüster et al. 2022). Due to the plasma-based mechanical damage to the hollow organs and the supposed multimodal mode of action, resistance is unlikely, in contrast to environmental chemical acaricides or acaricidal drugs (Heinlin et al. 2010). The use of CAPP as an alternative physical control method, if successfully transferred into practice, may help to reduce the use of environmental chemical acaricides and acaricidal drugs to control PRM in laying hen houses (Rüster et al. 2022). As a chemical-free method, CAPP could make a valuable contribution to animal health by reducing the number of infections requiring treatment and improving housing conditions, thereby improving animal welfare. Results to date have shown that even short-term application of CAPP is effective against PRM (Rüster et al. 2022). This cold plasma technology for practical use in occupied laying hen flocks is currently under development. The plasma system is designed as a box that can be used by the mites as a shelter and hiding place during the light phase, based on mite traps with a corrugated cardboard structure (Rüster et al. 2022). The plasma system can be used in close proximity to the host without the chickens coming into direct contact with the plasma technology (Rüster et al. 2022). This could be achieved, for example, by placing the box on the PRM’s path to or from the host, e.g. under the perches. The method could be adapted to current modular housing systems without major structural changes. Further research is needed to clarify the possible influence of stable climatic parameters such as dust pollution, humidity, noxious gases such as ammonia or hydrogen sulfide, etc. on the functionality of the plasma sources (Rüster et al. 2022). In addition, it is necessary to determine how many plasma systems are required depending on the size of the house, the housing system and the number of laying hens in order to achieve a sustainable effect on the mite population. These questions can only be answered in a field study once practical devices for mite control in occupied poultry houses have been produced. In addition, the exact mechanisms of action of CAPP need to be further investigated in order to realize its full potential for pest control.

## Data Availability

No datasets were generated or analysed during the current study.
